# Genomic landscape and evolutionary dynamics of *mariner* transposable elements within the *Drosophila* genus

**DOI:** 10.1186/1471-2164-15-727

**Published:** 2014-08-27

**Authors:** Gabriel Luz Wallau, Pierre Capy, Elgion Loreto, Aurélie Hua-Van

**Affiliations:** Pós-Graduaíão em Biodiversidade Animal, Universidade Federal de Santa Maria, Santa Maria, Brasil; Departamento de Biologia, Universidade Federal de Santa Maria, Santa Maria, Brasil; Laboratoire Evolution, Génomes, Spéciation, UPR9034, CNRS, Gif-Sur-Yvette, France; Université Paris-Sud, Orsay, France

**Keywords:** *Drosophila*, Comparative genomics, *Tc1-mariner*, Transposable elements, MITEs, Deletion rate

## Abstract

**Background:**

The *mariner* family of transposable elements is one of the most widespread in the Metazoa. It is subdivided into several subfamilies that do not mirror the phylogeny of these species, suggesting an ancient diversification. Previous hybridization and PCR studies allowed a partial survey of *mariner* diversity in the Metazoa. In this work, we used a comparative genomics approach to access the genus-wide diversity and evolution of *mariner* transposable elements in twenty *Drosophila* sequenced genomes.

**Results:**

We identified 36 different *mariner* lineages belonging to six distinct subfamilies, including a subfamily not described previously. Wide variation in lineage abundance and copy number were observed among species and among *mariner* lineages, suggesting continuous turn-over. Most *mariner* lineages are inactive and contain a high proportion of damaged copies. We showed that, in addition to substitutions that rapidly inactivate copies, internal deletion is a major mechanism contributing to element decay and the generation of non-autonomous sublineages. Hence, 23% of copies correspond to several Miniature Inverted-repeat Transposable Elements (MITE) sublineages, the first ever described in *Drosophila* for *mariner*. In the most successful MITEs, internal deletion is often associated with internal rearrangement, which sheds light on the process of MITE origin. The estimation of the transposition rates over time revealed that all lineages followed a similar progression consisting of a rapid amplification burst followed by a rapid decrease in transposition. We detected some instances of multiple or ongoing transposition bursts. Different amplification times were observed for *mariner* lineages shared by different species, a finding best explained by either horizontal transmission or a reactivation process. Different lineages within one species have also amplified at different times, corresponding to successive invasions. Finally, we detected a preference for insertion into short TA-rich regions, which appears to be specific to some subfamilies.

**Conclusions:**

This analysis is the first comprehensive survey of this family of transposable elements at a genus scale. It provides precise measures of the different evolutionary processes that were hypothesized previously for this family based on PCR data analysis. *mariner* lineages were observed at almost all “life cycle” stages: recent amplification, subsequent decay and potential (re)-invasion or invasion of genomes.

**Electronic supplementary material:**

The online version of this article (doi:10.1186/1471-2164-15-727) contains supplementary material, which is available to authorized users.

## Background

*Mariner* is a Class II transposon (i.e., transposing through a cut-and-paste mechanism) belonging to the large *Tc1-mariner-IS630* superfamily of transposable elements present in almost all groups of living organisms. It was first discovered in *Drosophila mauritiana* [1], a species from the *melanogaster* subgroup. Since then, *mariner*-like elements (*MLEs*) have been identified in a wide range of species, primarily metazoans. *MLEs* form a particularly homogeneous group of transposable elements (TEs), as all full-length copies share a size of approximately 1.3 kb, terminal inverted repeats (TIRs) of 28–30 bp and a unique open reading frame (ORF) encoding a transposase of approximately 345 amino acids. As with other members of the *Tc1-mariner* superfamily, transposases of the *mariner* family contain the typical catalytic DDE motif, which is necessary for transposition [2, 3]. However, in the *mariner* family, the DD(35)E signature is replaced by DD(34)D. Other conserved motifs with known (DNA-binding domain) or unknown functions have also been identified [4].

Based on the transposase phylogeny, the numerous *MLEs* can be grouped into several distinct subfamilies. Typically, *MLEs* within one subfamily share at least 40% identity at the amino acid level and between 40 and 56% at the nucleotide level [5]. Historically, the term *MLE* was used to designate any sequence showing sequence similarities with the original *mariner* elements (*peach* and *Mos1* copies), including consensus sequences derived from independent, closely related clones identified in the same species [6, 7]. The ability to derive a consensus sequence illustrates that the copies share a recent common ancestral copy (presumably similar to the consensus sequence) and constitute a (monophyletic) phylogenetic clade. It is assumed that within such a clade, any one mobilizable copy can be cross-mobilized by the active transposase of another copy, such as the inactive but full-length *peach* copy in *D. mauritiana*, which excises using the transposase of the *Mos1* copy [8]. These two copies are 99% identical at the nucleotide level [9] and belong to the same functional clade (usually referred to as *Dmmar1*, from the *mauritiana* subfamily of *MLEs*, and hereafter called *Dromar*1). *Mariner* clades are sometimes referred to as ‘types’ [10], ‘tribes’ [11], or ‘lineages’ [12]; this latter terminology will be used hereafter. Hence, the *mariner* family is composed of several subfamilies, each of which comprises several lineages.

While cross-mobilization is the rule within a lineage, copies from two different subfamilies are generally not expected to cross-mobilize because their nucleotide sequences will differ sufficiently such that the transposase from one lineage cannot recognize the TIRs of the other. *In vitro* experiments revealed that a 16% or more difference in the TIRs can preclude the binding of the transposase [13]. However, if the TIRs are similar enough, cross-mobilization may occur between two lineages from the same subfamily. Therefore, if two *mariner* lineages are sufficiently different, they may evolve and coexist independently within the same genome. Indeed, the coexistence of different lineages/subfamilies within the same genome is not uncommon [14, 15].

*MLEs* in animals are characterized by a patchy distribution, a high proportion of inactive copies and several suspected cases of horizontal transfer (HT). These properties led Lohe et al. [16] to propose a *mariner* lifecycle with HT as the starting point of the cycle. Amplification within the genome and the population is followed by diversification (vertical inactivation) and ultimately stochastic loss [16, 17]. In fact, this lifecycle may be a general rule for all transposable elements, as suggested by the recent theoretical studies of Le Rouzic et al. [18, 19]. The horizontal transfer step is the step under selection for the obvious reason that full activity is required for the transferred element to invade the new genome [20, 21]. However, once installed in a genome, TEs are subject to little or no selective pressure, as observed by Witherspoon and Robertson [14] in the *Caenorhabditis elegans* and *C. briggsae* genomes. In the absence of selection, mutations can accumulate, and this may explain the vertical inactivation.

Although *mariner* elements were first identified in *Drosophila*, a few different lineages have been found in this genus. Apart from *Dmmar1*, only two other lineages from the *mellifera* and *irritans* subfamilies have been found in *D. erecta* and *D. ananassae,* respectively, in studies aiming to identify *MLEs* in a wide range of insects and arthropods [5, 16]. One relic copy has also been identified in *D. melanogaster* [22]. In the present study, these elements are referred to as *Dromar6* (*D. erecta*), *Dromar5* (*D. ananassae*) and *Dromar14* (*D. melanogaster*). In the Drosophilidae, the *Dmmar1* (*Mos1*) lineage is the most thoroughly investigated lineage [23–26]. A wide range of species displays some hybridization signal (Southern blot or dot blot), which has been confirmed by sequencing in only some species, potentially reflecting the existence of other lineages from the *mauritiana* subfamily. A very patchy distribution has been found in the Drosophilidae, resembling the pattern observed at the larger scales of arthropods or metazoans. However, more recently, Wallau et al. [27] detected various *MLEs* from the *mellifera*, *mauritiana* and *irritans* subfamilies in 23 neotropical drosophilid species. Hence, *MLEs* are also diversified in the Drosophilidae.

**Figure 1:**
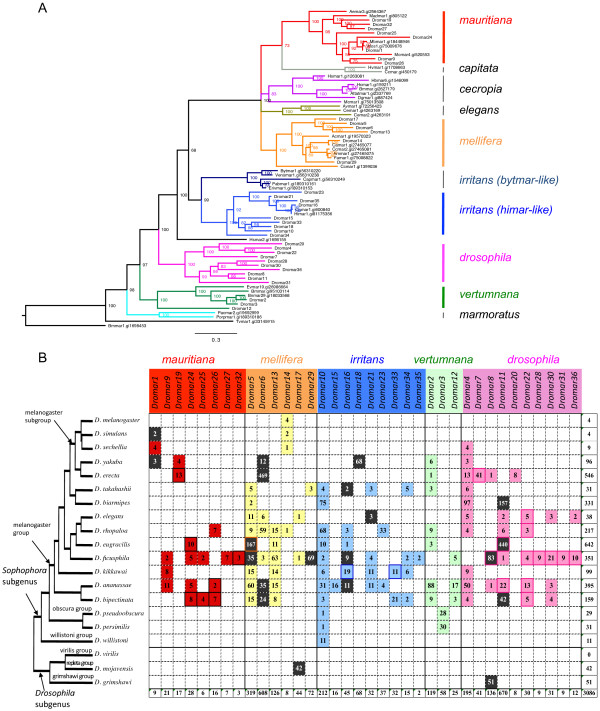
***mariner*** **lineages identified in this study. (A)** Phylogeny of *mariner-*like transposases generated by a Bayesian analysis using the WAG + G amino acid substitution model. Posterior probability of each node is indicated. Clade colors denote the different subfamilies of *MLEs* (indicated to the right of the tree)*.* The NCBI accession numbers of the transposases found in the database are indicated after the element names. **(B)** Distribution of MLE lineages in *Drosophila* genomes, indicating the number of independent copies, not truncated by assembly. Subfamilies are colored as in A. Dark colors indicate lineages with potentially coding copies. Framed boxes indicate lineages with MITEs.

The recently sequenced *Drosophila* genomes by the *Drosophila* 12 Genomes Consortium [28] and the *Drosophila* modENCODE project (Piano and Cherbas [29], http://www.genome.gov/Pages/Research/Sequencing/SeqProposals/modENCODE_ComparativeGenomics_WhitePaper.pdf) offer a unique opportunity to investigate i) the diversity of the *mariner* family in a group of both closely and distantly related species of the *Drosophila* genus, and ii) the evolutionary history of related but independent elements (see Figure 1B for a phylogeny of these species).

The *Drosophila* genus is separated into two subgenera: *Sophophora*, which contains *D. melanogaster*, and *Drosophila.* They diverged approximately 47 Mya ago, according to TimeTree2 [30]. Each subgenus is composed of multiple groups and subgroups, but *Sophophora* is most represented among the 20 sequenced genomes, with 17 species *versus* 3 for the *Drosophila* subgenus.

An exhaustive homology-based search in the 20 genomes recovered 36 lineages of *MLEs*, most of them not described previously. Phylogenetic analysis suggested that the 36 lineages belong to 5 different subfamilies, including one not identified previously. Although the genome dataset is highly biased toward species from the *melanogaster* group (*Sophophora* subgenus), comparison of these different *mariner* lineages within the different species provides a view of this TE family within one genus and offers clues to the evolution of *MLEs* by revealing *mariner* elements at each stage of their life cycle.

## Results

### MLE distribution and diversity within the genus *Drosophila*

**Figure 2:**
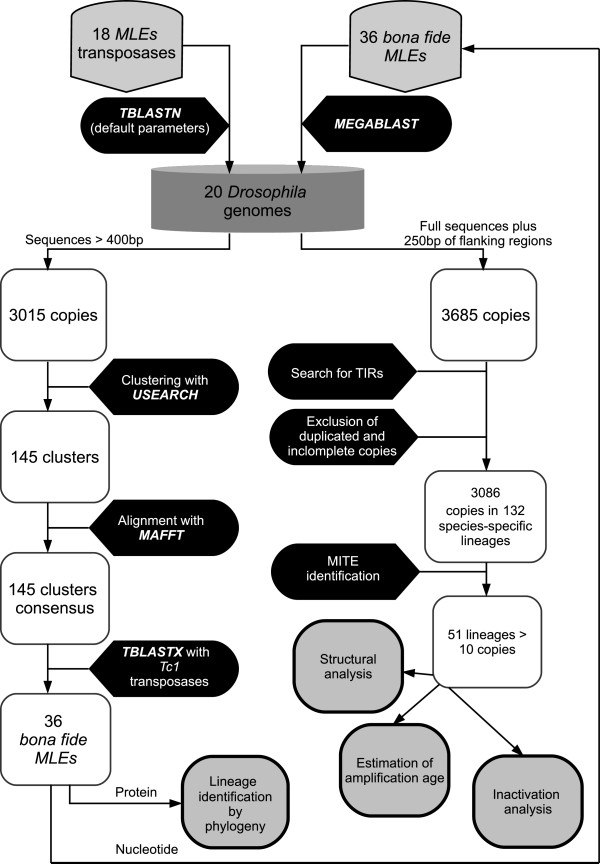
**Experimental design procedure showing all steps of the analysis.** We first searched by TBLASTN all 20 *Drosophila* using a set of 18 *MLEs* transposases representative of all described subfamilies of the *mariner* family. All sequences longer than 400 bp were clustered with a threshold of 80% identity. A consensus of each cluster was blasted against a transposase database composed of *Tc1* family elements to exclude sequences from this family. For the remaining 36 clusters (*bona fide MLEs*), consensus conceptual translations were used in a phylogenetic analysis together with the *MLEs* transposases, and the nucleotide consensus were used as queries in a MEGABLAST search in the same 20 genomes to correctly identify all copies. All hits plus 250 bp of flanking regions were retrieved. Structural and evolutionary analyses were performed on a clean dataset from which duplicated copies (segmental duplications) and incomplete copies (ends of contigs) were excluded.

A panel of 18 transposase sequences spanning eight of the main known *mariner* subfamilies (Table 1) were used in a TBLASTN search against the 20 *Drosophila* sequenced genomes (Table 2). The results were analyzed using an automatic procedure to separate the elements into different lineages and recover full sequences, including TIRs (see Methods and Figure 2). We identified 3685 copies corresponding to 36 different *MLE* lineages that we named *Dromar*1-*Dromar*36 (Additional file 1 and Additional file 2).

**Table 1:** ***mariner*** **transposases used in the first TBLASTN search**

	GI prot	UNIPROT	Subfamily (clade)	Species
*Mos1*	75009676	Q7JQ07_DROMA	*mauritiana*	*Drosophila mauritiana*
*Madmar1*	805122	Q25436_MAYDE	*mauritiana*	*Mayetiola destructor*
*Mcmar1-1*	75013508	Q869A8_MELCH		*Meloidogyne chitwoodi*
*Hsmar1*	1263081	Q13579_HUMAN	*cecropia*	*Homo sapiens*
*Dtmar1*	887424	Q24693_DUGTI	*cecropia*	*Dugesia tigrina*
*Avmar1*	72256423	Q45FI1_9BILA	*elegans*	*Adineta vaga*
*Cemar2*	7331821	Q9N523_CAEEL	*elegans*	*Caenorhabditis elegans*
*Cemar1*	7331903	Q9N4X9_CAEEL	*elegans*	*Caenorhabditis elegans*
*Avmar1*	72256423	Q45FH8_9BILA	*elegans*	*Adineta vaga*
*Ccmar1*	1399036	Q17312_CERCA	*mellifera*	*Ceratitis capitata*
*Acmar1*	19570323	Q8T0Y0_APICE	*mellifera*	*Apis cerana*
*Camar1*	27465077	Q8I8E8_CHYAM	*mellifera*	*Chymomyza amoena*
*Ccmar2*	27465081	Q8I8E6_CERCA	*mellifera*	*Ceratitis capitata*
*Famar1*	75008822	Q6XLA0_FORAU	*mellifera*	*Forficula auricularia*
*Hsmar2*	1698455	Q13539_HUMAN	*irritans (Hsmar-like)*	*Homo sapiens*
*Cpmar1*	600840	Q04514_CHRPL	*irritans (Himar-like)*	*Chrysoperla plorabunda*
*Bytmar1*	56310220	Q5QT23_9EUCA	*irritans (Bytmar-like)*	*Bythogtaea thermydron*
*Vesmar1*	56310238	Q5QT20_9CRUS	*irritans (Bytmar-like)*	*Ventiella sulfuris*

The historical *irritans* subfamily is divided into three clades following Bui et al. [31].

**Table 2:** **Characteristics of the genome assemblies used in this study**

	Scaffold number	Size (Mb)	WGS accession	Assembly
*D. melanogaster*	15	139	AABU01	Dmel_caf1
*D. simulans*	10,601	137	AAGH01	Dsim_caf1
*D. sechellia*	14,730	166	AAKO01	Dsec_caf1
*D. yakuba*	8,122	165	AAEU02	Dyak_caf1
*D. erecta*	5,124	152	AAPQ01	Dere_caf1
*D. takahashii*	1,792	182	AFFI02	Dtak_2.0
*D. biarmipes*	1,136	169	AFFD02	Dbia_2.0
*D. elegans*	1,063	171	AFFF02	Dele_2.0
*D. rhopaloa*	4,435	197	AFPP02	Drho_2.0
*D. eugracilis*	955	156	AFPQ02	Deug_2.0
*D ficusphila*	1,284	152	AFFG02	Dfic_2.0
*D. kikkawai*	1,284	164	AFFH02	Dkik_2.0
*D. ananassae*	13,749	231	AAPP01	Dana_caf1
*D. bipectinata*	1,552	167	AFFE02	Dbip_2.0
*D. pseudoobscura*	2,661	152	AAFS01	Dpse_caf1
*D. persimilis*	12,838	188	AAIZ01	Dper_caf1
*D. willistoni*	14,838	235	AAQB01	Dwil_caf1
*D. virilis*	13,530	206	AANI01	Dvir_caf1
*D. mojavensis*	6,841	193	AAPU01	Dmoj_caf1
*D. grimshawi*	17,440	200	AAPT01	Dgr0_caf1

The protein sequences translated from the 36 nucleotide consensus sequences were aligned with 36 other *mariner* transposases and subjected to phylogenetic analysis (Figure 1A). This analysis revealed that 26 *MLE* lineages belonged to 4 known subfamilies (*mauritiana*, *mellifera*, *irritans* and *vertumnana*). The 10 remaining lineages grouped together into a highly supported clade that appears to correspond to a new subfamily, which we designated *drosophila.* The clade structure derived from the transposase analysis was supported by the comparison of TIRs, which showed that some blocks of nucleotides are clade (subfamily)-specific (see Additional file 3).

For each lineage, we analyzed flanking regions to filter out duplicated copies (segmental duplication) and retain only independent copies resulting from transposition. All truncated copies located at contig ends were also discarded. This resulted in a clean dataset of 3085 copies, corresponding to 132 different *MLE*/species lineage combinations that were used in all further analyses (Figure 1B). The copy number varies from 1 to 469 (*Dromar6* in *D. erecta*) per lineage per species. In the *mauritiana* subfamily, all lineages presented a low copy number ranging from 1 to 13, in agreement with what was observed previously for the historical *Dmmar1* (*Dromar1*) lineage in *D. mauritiana* [1], which does not exceed 20 copies. In contrast, higher copy numbers were observed in other subfamilies. For instance, with 1142 independent copies and 10 different lineages, the *Drosophila* subfamily appears to be the most successful in *Drosophila*. This large variation may reflect some subfamily-specific properties.

On average, six MLE lineages coexist in the same genome. However, large differences are observed among species. Globally, the subgenus *Drosophila,* composed of *D. grimshawi*, *D. virilis* and *D. mojavensis,* appears to be poor in *MLEs* (only two lineages identified). Indeed, in *D. virilis*, no *mariner* was detected in a BLASTN search using the 36 *mariner* sequences with default parameters. The search with blastn-short option detected one fragment < 500 bp, which was distantly related to *Dromar18*. Therefore, this genome appears to be prone to eradication of such elements. Only one lineage was present in each of *D. grimshawi* and *D. mojavensis*. However, in both cases, these elements presented a substantial copy number (51 and 42 independent copies, respectively, totaling 90 and 52, respectively; see Additional file 1), most of them very similar. In addition, they included potential autonomous elements, which suggests a recent origin (see below). In the subgenus *Sophophora*, all species contained at least traces of *MLEs* and, with the exception of *D. melanogaster* and *D. willistoni*, contained at least two different *MLE* lineages. In *D. erecta* and *D. yakuba,* 8 and 6 lineages, respectively, were found, representing 4 and 5 different subfamilies, respectively. This diversity is particularly prevalent in *D. ananassae* and *D. ficusphila,* where the 5 subfamilies are represented by 18 and 23 lineages, respectively.

Nine MLEs lineages are restricted to only one species. Among the 33 lineages detected in the *melanogaster* group (*Sophophora* subgenus), twelve are subgroup-specific, and twenty-one are shared by two or more subgroups of species. Only two lineages are also present in other groups of the same subgenus (*Dromar10* in *D. melanogaster*, *D. obscura* and *D. willistoni*) or in species from the *Drosophila* subgenus (*Dromar8*) (Figure 1B). Hence, large differences are observed among species in copy number and diversity of MLEs, which may reflect specific properties of the genome or merely the independent evolutionary history of these lineages.

### MITEs arising from internal deletion and/or rearrangement

**Figure 3:**
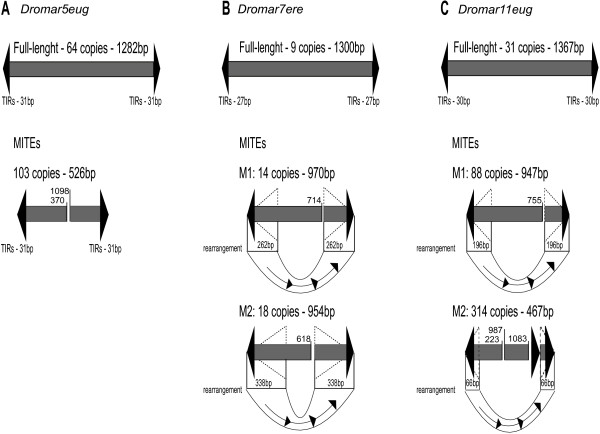
**Schematic representation of different MITEs and their progenitor full-length elements. (A)** *Dromar5eug* identified in *D. eugracilis* **(B)** *Dromar7ere* from *D. erecta.* **(C)** *Dromar11eug* from *D. eugracilis*. The rearrangements, which consistently involved the replacement of the 3′ region of the element with the 5′ region, are depicted below the elements. The sizes of the rearranged fragments are indicated and correspond to the size of the new inverted terminal repeats, shown as dashed triangles. Numbers above elements correspond to the position of breakpoints relative to the full-length element.

Short non-autonomous copies are often found among Class II elements. The amplification of such short copies can ultimately lead to the emergence of a MITE (Miniature Inverted repeat Transposable Element). MITEs are present in numerous species and can be very abundant, but to date, few MITEs have been identified in *Drosophila* species or within the *mariner* family. Non-autonomous short copies that have transposed at least once (i.e., are present in at least two independent copies) were identified in fourteen *MLE* lineages, primarily from the *mauritiana* and *drosophila* subfamilies. A total of 27 independent sublineages could be distinguished, as one MLE lineage can derive more than one MITE sublineage in different species (e.g., *Dromar22* and *Dromar11*) or within the same species (e.g., *Dromar7* and *Dromar11*). Each sublineage was characterized by a specific independent deletion/rearrangement pattern, illustrating its origin from a single copy (see Figure 3).

In total, MITEs represent 724 copies, corresponding to 23% of all *mariner* copies. The copy number of full-length *mariner* was generally low except in a few lineages in which hundreds of copies are found. Ten of 27 MITE sublineages contained 10 or more (up to ca. 300) amplified short copies (see Additional file 4). One third of the lineages, mainly characterized by a low copy number, had no larger relatives in the genome, and most were species specific (except for a few copies in *Dromar22* that are shared by more than one species). They likely represent aborted MITE amplifications due to the loss of the autonomous partner.

The typical size of MITEs varied between 900 and 1000 bp; however, in four lineages, copies were approximately 460–560 bp (Additional file 4). The two most successful MITEs are the ones with the shortest sizes.

For all MITE families, internal sequences and TIRs were fully homologous to the sequences of the full-length copies, with no traces of long, non-homologous sequences. Indeed, most of the MITE lineages corresponded to internally deleted elements; for example, *Dromar5eug* contains 112 MITE copies of 526 bp and 63 full-length copies (Figure 3A). This lineage is widely distributed across the *melanogaster* group, with a high copy number not only in *D. eugracilis* but also *D. ananassae* (77 copies). However, the MITE sublineage was specific to *D. eugracilis*.

Seven MITE sublineages from the *drosophila* subfamily clearly originated from rearrangements: two for *Dromar7* in *D. erecta*, three for *Dromar11* in *D. eugracilis* and one each for *Dromar28* and *Dromar36*. All resulted from the replacement of the 3′ portion by the 5′ sequence (Figure 3B, C). These rearrangements cause these copies to have unusually long TIRs. Hence, internal rearrangements appear to be a major process involved in the generation of MITEs.

MITEs have been associated with genes in plant species [32, 33]. We investigated whether this association was present in the high-copy-number *Dromar11* and *Dromar5* MITEs in *D. eugracilis*. Because this genome is not yet annotated, we first identified potentially coding regions using a TBLASTN search with the protein database of *D. melanogaster* and then computed the distance of *Dromar11eug* and *Dromar5eug* copies to these potential CDSs (Additional file 5). For both lineages, the proportion of copies present in the same supercontig as a putative CDS was higher for MITEs than for non-MITE copies. However, for copies present in a CDS-carrying contig, we could not detect significant differences in the distance-to-CDS distribution of MITE and non–MITE copies (KS test). Nevertheless, a significant difference was observed between *Dromar5Meug* and *Dromar11M1eug*, as well as between *Dromar5* and *Dromar11* lineages overall. These two lineages differ in age, with *Dromar11* being more recent (see below); its copies were also globally closer to CDS, which suggests that older lineages are farther from genes. This finding equally supports two hypotheses: long-term selection against insertion near genes *versus* the preferential insertion of new copies near genes pushing away old insertions.

These MITE families were separated from their associated full-length elements for subsequent analyses.

### Mobility and coding potential in each lineage

**Figure 4:**
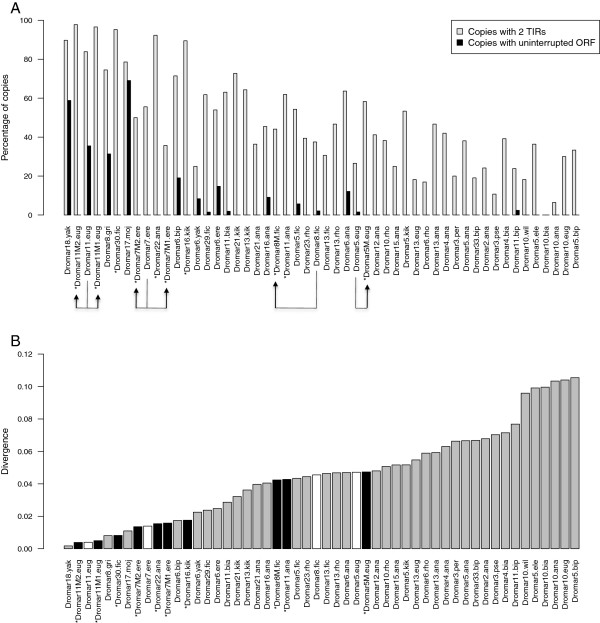
**Structural characteristics of** ***mariner*** **lineages copies. A** - Proportion of copies at two different conservation stages: with 2 TIRs and with an uninterrupted ORF between 330 and 363 AA in all lineages with at least 10 independent copies. An asterisk before the name denotes MITE sublineages. Arrows indicates full-length lineages and the derived MITEs sublineages. **B** - Mean divergence of the same lineages relative to the consensus sequence. Black bars are MITE sublineages, whites bars are their full-length partners.

In each species, for each lineage with at least ten copies (52 species-*mariner* lineages representing a total of 2673 copies), we counted the number of i) potentially mobilizable copies (characterized by two full-length TIRs), and ii) potentially coding copies (i.e., presenting an uninterrupted ORF of the expected size of between 330 – 363 aa). These various (non-mutually exclusive) types of copies may provide information on how MLEs decay over time; i.e., through loss of transposition ability or loss of activity. However, this crude analysis is likely to yield an overestimation of the number of active copies, particularly in old lineages, as substitutions in the sequence may lead to inactivation of the transposase without affecting the size of the ORF. In addition, we characterized the divergence of these lineages relative to their respective consensus sequence. Although it may be biased by the structuration of the lineage, the divergence from the majority-rule consensus is often used as an estimate of lineage age (Figure 4A, B).

First, we observed that only 15 lineages contained copies with an uninterrupted ORF. Four lineages containing between 20 and 50% of potentially active copies also corresponded to the less diverged lineages (less than 2%). In less conserved lineages, the proportion of copies with an uninterrupted ORF was typically very low, below 20%. Second, more than half of all copies (54%) still harbor both TIRs and may likely be mobilizable. Again, less diverged lineages approximately corresponded to those with more potentially mobilizable copies. MLE copies have previously been found to be frequently inactive due to frameshifts and stop codons [16, 26], which our analyses confirm. The loss of TIRs by deletion also appears to be important in explaining the decay of a lineage. *mariner* lineages appear to inactivate rapidly through mechanisms that impair either the coding ability of copies (modification of the ORF) or its ability to move (modification of the TIRs).

The few lineages with a high proportion of potentially active copies and a high proportion of 2-TIRs copies also contain a high number of copies, which likely reflects recent amplification activity. However, all other combinations were also observed, and no correlation could be established among copy number, mean divergence time and the presence of functional ORF or TIRs. Hence, some lineages are recent and active but with few copies (e.g., *Dromar1*, for which activity is biologically demonstrated); others are recent but inactive with few copies (e.g., *Dromar9* and *Dromar7*). Others are ancient with many relic copies (e.g., *Dromar2* and *Dromar10*). This analysis illustrates that each lineage has its own evolutionary history and that no one rule applies, even within the same genome.

Finally, among recent (less diverged) lineages, we found seven of the ten MITE sublineages included in our analysis. However, only two (*Dromar11M1eug* and *Dromar11M2eug*) were associated with a full-length lineage with potentially active copies in the same genome. Two others (*Dromar5Meug* and *Dromar8Mfic*) also have potentially active partners in the genome, but belong to older lineages. For the four “orphan” MITE lineages with no full-length partners (*Dromar30fic*, *Dromar11ana*, *Dromar22ana*, and *Dromar16kik*), we could not identify close active lineages that might have provided the transposase needed for transposition. However, we cannot exclude the possibility that active copies still exist in other individuals or populations of the species.

### Mechanisms of inactivation

To determine whether some processes of inactivation are more prevalent than others, , we calculated the number of nucleotides lost, the number of nucleotides gained by insertion and the number of substitutions for each of the 52 species-*mariner* lineages. For indels, we distinguished cases in which deletion encompassed TE sequences only (internal deletions, potentially impairing ORF) from those extending into flanking regions, in which one end of the TE is lacking (truncation, affecting at least the mobility of the copy). A large insertion, alone or followed by other rearrangements, may also lead to the apparent truncation of the copy.

**Figure 5:**
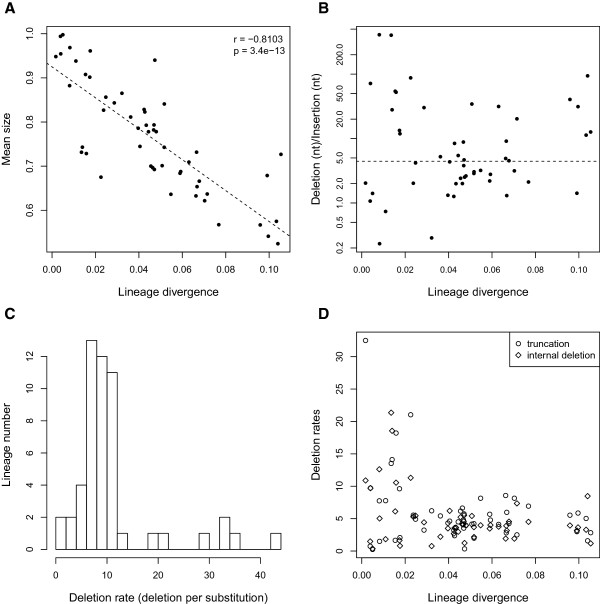
**Degeneration patterns observed for lineages with at least 10 independent copies. (A)** Mean size of copies in each lineage as a function of lineage divergence. The dotted line indicates the linear regression. **(B)** Ratio of nucleotides lost (by truncation or internal deletion) to nucleotides gained by insertion (log scale) as a function of lineage divergence. The dotted line corresponds to the median ratio. **(C)** Histogram of the deletion rate (number of nucleotides lost per substitution per lineage) distribution. **(D)** Deletion rates (internal deletion rate and truncation rate are dissociated) as a function of lineage divergence.

The first graph (Figure 5A) plots the average size of copies against lineage divergence (both relative to the consensus, excluding insertion). In agreement with the results shown in Figure 4, the observed negative correlation indicates that size reduction is a continuous process that, along with substitutions, contributes to the aging, inactivation and potentially, the disappearance of the lineages. Indeed, up to half of the original nucleotides can be lost in old lineages, ignoring the too-short copies (<400 bp) discarded during the initial search. Counting the insertions only reduces the coefficient of correlation (−0.7656), indicating that nucleotide gain by insertion cannot offset the number of nucleotides lost by internal deletion or truncation. Indeed, the number of nucleotides lost by internal deletion almost always exceeded the number gained by insertion, regardless of lineage age (mean divergence) (Figure 5B). The same pattern was revealed when computing the number of events (not shown, see Additional file 6). However, most internal deletion events are of very small size. The average size of deletions did not exceed 102 nt, and the maximum median deletion size was 22 nt (Additional file 6).

The ratio of deleted nucleotides to substitutions (Figure 5C) ranged from 1.5 to 45, but was between 4 and 12 for most lineages. This indicates that deletion is consistently more prevalent than substitution and is thus a major process leading to family inactivation. Plotting this ratio against nucleotide divergence (as a measure of lineage age) suggests that this may be particularly true for old lineages, whereas young lineages display more variable, sometimes very high, ratios (Figure 5D). A likely explanation for this pattern is that the high variability in young lineages is due to stochastic effects (occurrence of rare large deletions or truncations), whereas in old families, the effect of large deletions is offset by a higher number of substitutions. Alternatively, multiple large deletions in an old lineage will lead to the disappearance of copies and subsequently the lineage itself.

### Amplification age and evolutionary history

**Figure 6:**
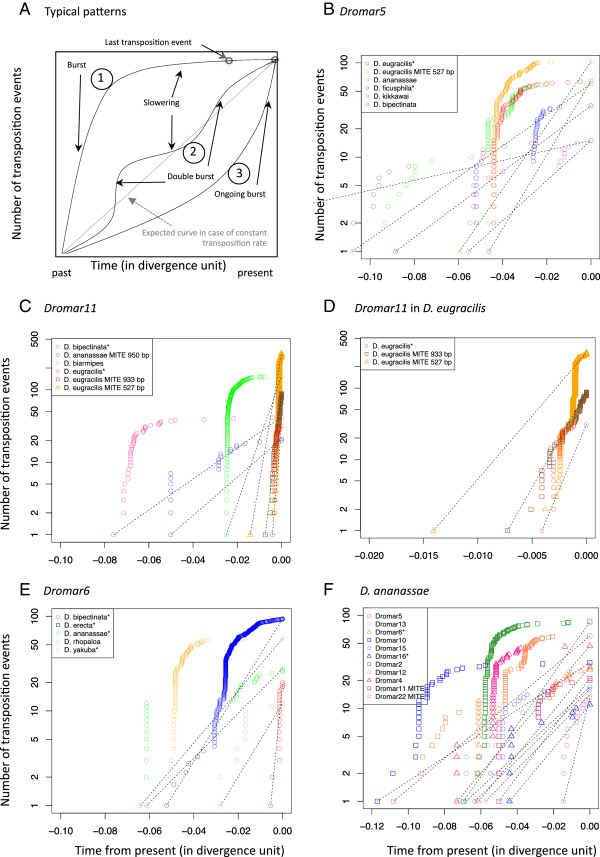
**Dynamics analysis of different lineages in different species. (A)** Interpretation of curve shape in theoretical LTT (Lineage-Through-Time) plot (cumulative number of transposition events over time, measured in genetic divergence units). **(B)** LTT plot of *Dromar5* in 5 species. The dotted lines represent the theoretical curves assuming a constant transposition rate per copy over time (exponential transposition). **(C)** Same analysis as in B for *Dromar11*. **(D) -** Magnification of **C** for the three recent *D. eugracilis Dromar11* sublineages. **(E)** Same analysis as in B for *Dromar6.* **(F)** LTT plot of different lineages in *D. ananassae*. Colors reflect the subfamily of each lineage following the color code in Figure 1.

In all our analyses, deletions affect copy functionality in an easily detectable way (ORF shortening and frameshifts), whereas substitutions leading to inactivation are only visible when they create a stop codon. Hence, among lineages that have apparent coding potential, some may actually be inactive. The divergence from consensus measure provides indications of the age of the lineages and the time of expansion, but it does not reveal whether the lineage remains active. Furthermore, this measure is only accurate when the consensus sequence reflects the ancestral active sequence. To estimate the global dynamics and activity of *mariner* lineages, we used the methodology developed by Le Rouzic, Payen and Hua-Van [34], based on phylogeny. In the tree, each node corresponds to a duplicative transposition and can be computed in a Lineage-Through-Time (LTT) plot depicting the rate of duplicative transposition events through time (Figure 6A). By this method, the variation in transposition rate per copy over time can be readily evidenced and amplification burst(s) within a species can be dated (in divergence units), allowing comparisons among species or *MLE* lineages. More simply, the evolutionary pattern can be inferred from the shape of the curve. Three schematic patterns are shown in Figure 6A that reflect the major patterns that arise from the analysis of the main *mariner* lineages. The first pattern is a past amplification burst followed by a stabilization in copy number, suggesting a loss of activity. The second pattern reveals successive amplification bursts. The third pattern involves an increase in transposition rate with time, compatible with an ongoing burst.

We used this methodology to analyze (i) the same lineages in different species and (ii) different *mariner* lineages within the same species.

We first focused on some lineages exhibiting a large distribution, a high copy number and some potentially active copies (in *Dromar5*, *Dromar6* and *Dromar11*). *Dromar*5 is present in all species from the *melanogaster* group with the exception of the *melanogaster* subgroup. This suggests that it was present in the common ancestor of this group, an inference supported by the presence of ancient copies and ancient bursts detected in some species (Figure 6B). However, in each species, the lineage amplified at different time (assuming a comparable molecular clock among all species). This lineage has been particularly successful in *D. eugracilis*, with numerous copies including a MITE family, and in *D. ananassae* and *D. ficusphila*. In *D. eugracilis*, MITE and non-MITE copies amplified at the same time. However, the non-MITE lineage has continued amplifying more recently than the MITE lineage. In *D. ananassae* and *D. bipectinata*, the lineage appears to be extinct; it also appears to be extinct in *D. ficusphila*, despite the presence of a potentially active copy in this species. In contrast, the lineage may not be totally extinct in *D. kikkawai*, although no potentially active copy has been detected in the sequenced genome.

*Dromar11* is present in several species from the *melanogaster* group, and MITE lineages have been detected in *D. eugracilis* and *D. ananassae*. Again, amplification appears to have occurred at different times in each species (Figure 6C). An ancient transpositional burst followed by a decrease in activity is apparent in three species: *D. biarmipes*, *D. bipectinata*, and *D. ananassae.* The lineages may not be completely inactivated, as recent transpositions were also detected, compatible with the presence of potentially active copies in *D. biarmipes* and *D. bipectinata*. However, this is not expected for *D. ananassae*, in which only a MITE sublineage persists. In *D. eugracilis*, the lineage amplified more recently. For the two MITE sublineages, amplification is clearly ongoing (Figure 6D). However, it appears to have slowed down for the non-MITE partners. This situation illustrates the competition that may occur between autonomous and non-autonomous elements [35].

*Dromar6* contains potentially active copies in four species. The analysis shown in Figure 6E revealed that the lineage is still transposing in these four species and that it is inactive in *D. rhopaloa*, which contains no potential active copies. In *D. bipectinata*, a species closely related to *D. ananassae*, the lineage appears recent and in an ongoing burst; this may be explained by recent acquisition through horizontal transfer.

Figure 6F illustrates the dynamics of different *mariner* lineages within *D. ananassae*, which contains numerous lineages. Among the 11 lineages analyzed, two old ones (*Dromar10* and *Dromar5*) appeared to be completely extinct, following one or more transposition periods (double burst). Three displayed recent transposition: the MITE lineage *Dromar22*, which is the most recent in the genome but has no autonomous partner, and the two lineages with potentially active copies that have suffered several transposition bursts, suggesting reinvasion or reactivation of lineages. Indeed, these lineages do not appear to be more recent than several other lineages that are now extinct, and they first amplified during the same period (0.4 to 0.6 divergence units from present). Hence, amplification of MLE lineages occurs regularly, with each transposition burst eventually replaced by another one from another lineage.

### TA insertion sites are not random

**Figure 7:**
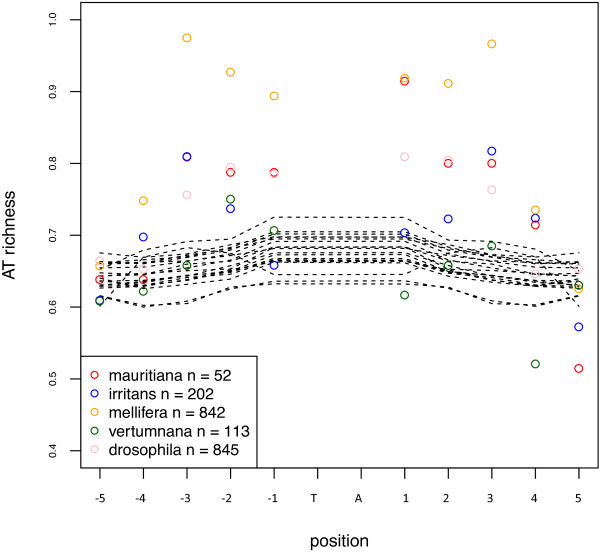
**Percentage of TA at each position around the insertion TA.** Results for each subfamily are shown as colored open circles (n: number of analyzed TA) and are compared to that obtained for all TAs in the 20 genomes (dotted black lines).

*Mariner* elements, like all elements of the *Tc1-mariner- IS630* superfamily, insert into TA dinucleotides that are duplicated upon insertion. Few studies have searched for preferences other than this strict target site. To determine if there are differences in the immediate environment between insertion and non-insertion sites, we analyzed the region spanning 5 nt on either side of the insertion sites of some lineages from the five subfamilies. The percentage of T and A at each position was compared to the average percentage across all TAs of the genome (Figure 7). Some biases were observed within the 3 nt on either side of the insertion. A strong bias was observed for the *mellifera* subfamily, which appears to insert in very TA-rich regions. A similar tendency was observed for *mauritiana* and *drosophila* copies. For *irritans*, a bias was observed at the −3 and +3 positions. An analysis of the preference of the *mellifera* and *drosophila* subfamilies for trinucleotides revealed that not all TA-rich sequences are used. For example, for *mellifera*, the sequence ATA is highly preferred over the sequence ATT in the 5′ end of the insertion site, although in this case, we did not compute the frequency of these trinucleotides across the entire genome (Additional file 7). A WebLogo analysis confirmed the existence of a consensus sequence composed of an AT stretch for the *mellifera, drosophila and mauritiana* lineages (Additional file 8). In contrast, in the *vertumnana* and the *irritans* subfamilies, no consensus could be detected. The presence of the consensus was independent of copy number, active status, or species, but may be affected by the age of the lineage in a species (e.g., *Dromar11* and *Dromar8*, which both have different amplification times between species). In this case, the apparent weakness of the consensus in old lineages may be explained by mutations that appeared after insertion. This suggests that the stringency of the TSD choice depends mainly on the peculiarity of each transposase, in terms of its binding affinity to or cutting efficiency of a particular sequence and is conserved within subfamilies.

## Discussion

### Methodology

The use of both TBLASTN with 18 query transposases and MEGABLAST allowed us to identify more than 3685 copies representing 36 different *mariner* lineages. TBLASTN provided several hits that were identified as *Tc1*-like sequences, which indicates that the search was likely to be exhaustive. However, non-autonomous lineages could have been missed because we did not recover too-short sequences (<400 bp) or sequences with no conserved transposase domain, generated by internal deletions. Copies interrupted by insertions of less than 1000 nt could be reassociated. However, if an insertion is longer than 1000 nt, one copy could appear as two independent truncated copies. However, this imprecision is not expected to strongly bias the results. Hence, the panel of retrieved copies can be considered as representative of the *mariner* panorama in the *Drosophila* sequenced genomes.

### Distribution, diversity and copy number across the genus

The 20 *Drosophila* genomes evaluated here belong mainly to the *Sophophora* subgenus and *melanogaster* group. Species from the same subgroup can have very different *mariner* content (e.g., *D. melanogaster* and *D. yakuba*). However, *mariner* lineages in the genomes of sister species appear more similar (e.g., *D. simulans* and *D. sechellia,* and *D. pseudoobscura* and *D. persimillis*). In this latter case, the majority of the copies lie at orthologous sites, allowing us to date the expansion of this lineage prior to speciation.

In the subgenus *Drosophila*, only 2 *mariner* lineages could be detected after the filtration process. This finding suggests that this subgenus is poor in *mariner* lineages. However, PCR searches for *mariner* elements in several species from the *Drosophila* subgenus have revealed the presence of at least three different subfamilies (*mellifera*, *irritans* and *mauritina*) in several neotropical species [27]. The low representation of *MLEs* in this subgenus may be due to poor sequencing sampling and the fact that those species are distantly related to those tested by Wallau et al. [27].

The 36 lineages identified across the *Drosophila* genus belong to 5 different subfamilies. One subfamily appeared as a new uncharacterized subfamily, and we named it the *drosophila* subfamily. However, representatives of this subfamily have been detected in a very distant genome (the cnidarian *Hydra magnipapillata*), which is described as a genome rich in *Tc1-mariner* in which several horizontal transfers have occurred [36].

Several of the 36 lineages are restricted to one or a few closely related species. Most of the lineages are in very low copy number and can therefore be easily lost. These findings suggest that the total number of different lineages within the *Drosophila* genus is large and that analyzing new species will uncover new lineages.

### *Mariner*activity and inactivation

In addition to the wide diversity of *mariner* lineages, most of them correspond to inactive lineages. The absence of potentially active copies does not mean that the lineage is extinct, as the data come from highly inbred lines and represent little of the variability in natural populations. Nevertheless, this result supports previous findings that *mariner* elements are easily vertically inactivated [16]. In the inactivation process, the loss of nucleotides appears to play an important role. In old lineages, almost half of the nucleotides are lost. Therefore, the fate of *MLEs* in a genome appears to be elimination. Complete removal of a lineage can occur through a persistently low level of reinsertion or vertical inactivation followed by rapid elimination by the genome. The large variety of *mariner* lineages in the *Drosophila* genomes, along with the deep phylogenetic relationships, high deletion rate (Figure 4) and very recent amplifications of these elements (Figures 4 and 6), suggest that this diversity is ancestral and that these genomes might be regularly fed with the old lineages through horizontal transfer events. The deletion rate data are in agreement with the high rate of deletion found for the *helena* non-LTR retroelement in the *D. melanogaster* and *D. virilis* genomes [37, 38]. Some *MLEs* presented a higher deletion rate than other *mariner* lineages, as is shown in Figure 4D. However, these lineages with high deletion rates are among the more recently amplified ones and were found in different species; therefore, it is not clear whether some deletion mechanisms are responsible or if this pattern is due to stochastic effects.

### MITEs

Originally, MITEs were described as high copy number, short elements in plants [39], reaching thousands of copies. In *Drosophila,* few MITEs have been characterized to date, and the copy number has not exceeded 100 [40–42]. Furthermore, few *mariner* MITEs have been described, the most notable case being the *MiHsmar1* family in humans [43]. Almost a quarter of the copies corresponded to 27 different MITE sublineages from 14 MLE lineages, 17 from the *drosophila* subfamily and 6 from the *mauritiana* subfamily, suggesting that these subfamilies were particularly likely to generate such shorter elements. The copy number was typically very low among both the MITE sequences and the longer elements. The term “MITEs” might therefore appear inappropriate, although these copies transposed at least once, as indicated by their flanking sequences. However, it cannot be excluded that these low copy number MITEs reached a high copy number, as is theoretically possible for the few lineages with high copy number (from 100 to more than 300), which has never before been observed in *Drosophila*.

The approximate size uniformity (23 sub-lineages between 900 and 970 bp, and 4 between 467 and 560 bp) was particularly unexpected and suggests that MITE transposition ability may be strongly constrained by either size or structure. This scenario is supported by the observation that in other internally deleted elements (that have not amplified), the size distribution does not exhibit any strong bias (not shown). Size constraints have been reported for some *mariner* elements [44], although there are also examples of successful transposition events of very short elements among MLEs [43, 45].

Although knowledge of the dynamics and the transposition process of MITEs continues to grow, the origins of these elements remain unclear. Indeed, sometimes the sequence similarities to the putative autonomous partner are restricted to the TIRs. In other cases, large TIRs are present, but similarities are only visible at the tip of the TIRs [46]. The *de novo* formation hypothesis (that similar solo-TIR-like sequences close to each other may generate these short non-autonomous TEs) has not found empirical support to date. The alternative hypothesis assumes an internal deletion of the autonomous element, with degeneration or substitution of internal sequence [40, 47]. Amplification could result from the recognition of TIRs by the transposase of active copies from the same lineage or from distantly related lineages [48]. In our study, all MITE lineages could be traced back to a putative *mariner* full-length lineage due to homologous parts in the internal region, as expected from the homology-based strategy used. No instance of severe degeneration was detected. Our analyses indicate that internal deletion appears to be a major process in the generation of non-autonomous copies and MITEs. However, we detected seven cases where internal deletion was accompanied (or caused) by rearrangements systematically consisting of the replacement of the 3′ part of the element by a 5′ portion. In one case, corresponding to the most abundant MITE, two TIRs were present in the 3′ region. The absence of MITE copies with only one 3′ terminal TIR indicates that the most external TIR is strongly favored for transposition, perhaps providing the element with optimal size. Optimal size may add further to the potential absence of a repressive motif in the MITEs, explaining MITE success [49]. A side effect of the rearrangement process is the large increase in the size of the inverted repetition, which may increase transposition ability by stabilizing the synaptic complex. Although experimental evidence is lacking, successful MITEs, such as the first ones identified in plants, exhibit a high potential to form stable secondary structures [50]. Finally, whereas the origin of internal deletions can easily be explained by abortive gap repair [51], the origin of more complex rearrangement remains elusive.

### Dynamics of transposition and the horizontal transfer hypothesis

The dynamics analysis revealed several patterns that conflict with the hypothesis of a constant transposition rate per copy. The temporal variation in transposition rate per copy appears to consistently follow the same progression: an initial high transposition rate corresponding to a burst of amplification, followed by progressive attenuation. This pattern may be due to two non-exclusive processes. First, copy inactivation acts rapidly, diminishing the proportion of active copies (encoding an active transposase). Therefore, if transposition is limited by transposase availability, the transposition rate per copy will decrease. Second, the decrease in transposition rate per copy over time may reflect the initiation of regulatory processes. The relative contributions of these processes can likely be estimated from the dynamics by considering active (encoding) and inactive (but *trans*-mobilizable) copies over time, which we aim to investigate in the future.

The similarities in the amplification dynamics suggests a general pattern, in accordance with theoretical models [52] and models derived from experimental observations [16]. However, the dynamics observed in other species and other Class II elements may be very different, such as those observed for *pogo*-like elements in the fungus *Fusarium oxysporum* [34]. In this latter case, transposition rates per copy do not vary widely over time.

*Mariner* is known to undergo frequent horizontal transfer (HT) [53]. Posited factors supporting HT are a patchy distribution, a TE phylogeny incongruent with that of the host and the presence of highly similar sequences in distantly related species [54]; this latter hypothesis is difficult to test definitely, as the true donor may not be part of the sample. Another possible factor that may reflect HT are differences in amplification times or patterns of the same lineage observed between different species.

Horizontal transfer followed by rapid amplification burst may explain why lineages appear as recent and active in some species, while old and inactive in others. *Dromar*8 displays a very patchy distribution, and it is present in distantly related species in which it displays varying amplification times. This lineage is very young in *D. grimshawi*, with a high proportion of potentially coding copies relative to the apparently older lineage present in the distantly related *D. ficusphila*. This lineage may represent a good candidate for horizontal transfer. *Dromar*18 and *Dromar*17 are two other recent lineages with a restricted species-specific distribution, and may also have arisen by HT. In particular, *Dromar18*, which is not present in closely related species, may have arisen by HT. *Dromar17* contains some old copies and may have resulted from a re-activation process.

However, several *mariner* lineages are widely distributed within the melanogaster group. Such a distribution suggests an ancestral presence and vertical transmission. Another hypothesis is that amplification of the lineage predated speciation within the group. However, the *Dromar5, Dromar6* and *Dromar11* lineages exhibited some amplification time differences among species, as did *Dromar4* (not shown), which suggests that the ancestral presence of the elements may not fully explain the observed pattern. Horizontal transfer with replacement of old copies or reactivation may have occurred. The detailed analysis of the amplification dynamics of *Dromar6* provides another example of a probable horizontal transfer. This lineage is present in the closely related species *D. ananassae* and *D. bipectinata*, and although this suggests its potential presence in the ancestor of the two species, the lineage in *D. bipectinata* latter species is much more recent and is therefore better explained by horizontal transfer.

As stressed previously [24], only a combination of these arguments constitutes a convincing basis for invoking HT. The results of the analysis of amplification time provide a new, additional argument. However, these arguments are insufficient to demonstrate HT, and further analyses are needed to test the HT hypothesis. One approach may include a new methodology for HT detection, which compares the synonymous substitution rates in elements and genes [20]; however, the results should also be interpreted with care [55]. We are currently developing a new method that automatically compares TEs and genes and is based on substitution rates and other parameters. This method may help detect HT at a comparative genomic scale.

### Competition at different levels

Transposable elements are pieces of selfish DNA; i.e*.*, DNA that promotes its own perpetuation in the genome without participating in the survival of the host cell or organism [56, 57]. Nevertheless, the exponential or infinite multiplication of TEs may be limited by their elimination through natural selection, once such multiplication becomes too harmful to the host [58, 59]. The establishment of regulatory mechanisms also explains why TE copy number may remain relatively low. Regulation by silencing through epigenetic processes is a universal strategy used by genomes to minimize the harmfulness of invading sequences [60]. The genome wide epigenetic silencing of TEs appears to result from the combination of processes targeted toward particular TE families and that rely on sequence homology. Hence, this host defense system is highly dynamic and illustrates the arms race between the genome and TEs [61]. Some authors have proposed an ecological view of the genome in which TE copies and families are viewed analogous to individuals and species that compete for the same ecological niches [19, 62, 63]. Competition between copies of the same lineage can occur when transposase resources become limited due to the progressive inactivation of TEs. At the extreme, some copies becomes parasitic on others, and MITEs constitute the most convincing example. The dynamics of *Dromar11* in *D. eugracilis* illustrates this process, as the full-length sub-lineage shows a marked lower transposition rate than the associated MITEs, at a recent time. As a counter example, the *Dromar5* full-length lineage appears to be more efficient in promoting its own transposition than was the associated MITE in the recent past. This finding may reflect that this older lineage is involved in a different stage of the cyclical interaction predicted by theoretical studies [35]. Further analysis of the mutational pattern may be useful in testing this hypothesis.

Parasitism and competition might even occur between copies from closely related lineages (same subfamilies) when they are able to cross-mobilize [49]. In genomes with numerous *mariner* lineages, there are several examples of concomitant amplification, suggesting that competition between lineages is limited. Nevertheless, we noticed that concomitant amplification never involved lineages from the same subfamilies. At the same time, several non-autonomous elements lack autonomous partners, but are recently amplified. No close partners could be identified, possibly because the sequenced genome of one individual, while potentially representative of the average genome of a population, does not contain every copy present in the population.

## Conclusions

With 36 different *mariner* lineages identified, this analysis sheds light on the powerful ability of *mariner* to diversify. We detected some subfamily specificity (low copy number for *mauritiana*, ability to form MITE sublineages for *mauritiana* and *drosophila*, strong insertion site bias for *mauritiana* and *mellifera*), although the causes remain unclear. The dynamics analysis revealed that amplification is the result of a short transposition burst followed by stagnation (and ultimately decay), most likely due to rapid inactivation or regulation. Horizontal transmission or reactivation may compensate for the continuous inactivation of copies that leads to the death of the lineage. This scheme is in accordance with the *mariner* life cycle suggested by Lohe et al. [16] from their analysis of MLE PCR fragments in various animals. The dynamics analysis also provides a way to access the competition process, particularly between non-autonomous and autonomous partners, previously evidenced in a theoretical study [19]. The different examples of MITEs detected here provide insights into the creation and amplification of these elements. This genus-scale analysis illustrates the power of comparative genomics to decipher the evolution of transposable elements. In the near future, accumulating population genomics data should permit deeper analysis and improved understanding of TE evolution.

## Methods

### Detection of *mariner*sequences

The strategy used to recover MLEs is depicted in Figure 2. We used several programs from the BLAST + suite [64]. Based on their availability in the protein database and their diversity, we selected 18 transposases (Table 1), covering at least 8 subfamilies, and used them as queries in a batch TBLASTN search performed on the WGS sequences of 20 *Drosophila* genomes [28] and Piano, Cherbas [29], using the default cut-off value. We recovered 14977 different hits (High Scoring Pairs) and reassembled them into 3694 copies according to the following criteria. Two hits from the same scaffold are grouped together if the distance between the center of each hit is less than 1000 bp and if they have the same orientation. Only copies longer than 400 pb were retained. Clustering with USEARCH v 6.0 [65] using a threshold of 80% identity provided 145 Clusters. A BLASTX search against transposases from the *Tc1* family was conducted with the consensus sequences of the 145 clusters as queries. Forty-six clusters were found to be more related to *Tc1* elements and were excluded from analysis. The remaining clusters were inspected manually. At the same time, a global alignment was performed with MAFFT v.7 [66] on the sequences and their flanking regions (250 bp each side). From these analyses, 36 clusters were considered *bona fide MLEs*, and consensus sequences were derived and translated into protein for phylogenetic purposes. Consensus sequences were also used as queries in a MEGABLAST search against the 20 *Drosophila* genomes to precisely determine copy numbers and copy ends (including truncated or deleted copies lacking recognizable conserved protein motifs). This resulted in 3685 sequences. The full sequence presenting homology along with 250 of each flanking sequences were then extracted. Duplicated sequences (same flanking regions) as well as truncated copies located at the end of contigs and supercontigs (ends of the sequence, presence of ‘N’ nucleotides) were further eliminated to obtain a clean dataset of 3084 sequences.

### Alignment and consensus

Nucleotides and proteins were aligned with MAFFT v.7 [66] or MUSCLE3.8 [67], and evaluated and refined by hand. Consensus sequences were derived using the relative majority rule. Gaps were distinguished between internal gaps and truncations. When the majority was an internal gap, a gap was included in the consensus. Gaps at the end of the sequences were not counted. Consensus sequences were evaluated and corrected as needed to obtain full-length sequences.

### Distance to genes

The distance of elements to genes was calculated for *Dromar5* and *Dromar11* in *D. eugracilis*. The *D. melanogaster* protein database was used for TBLASTN queries on the *D. eugracilis* genome to identify homologous gene sequences positions. The distance to the closest putative gene was then computed for copies present in the same contig as a gene.

### Inactivation analysis

Copies were assumed to contain 2 TIRs if they were not truncated at the end. Hence, mismatches or substitutions in the TIRs, which may affect mobility, were not accounted for. Copies were considered to possess an uninterrupted ORF if the ORF comprised between 330 and 363 codons. It is likely that this relaxed constraint leads to the overestimation of the number of such copies. Furthermore, the ability to encode an active transposase also depends on the protein sequence. The lineage divergence is based on the divergence to the consensus, recalculated for each lineage in each genome. It takes into account the total number of substitutions and the total numbers of insertion and deletion events. The sum is then divided by the total number of nucleotides aligning with the consensus sequence (large insertions are not counted). For indels, the numbers of nucleotides inserted and deleted were calculated relative to the consensus sequence. For deletions, two cases were considered: internal deletions or end truncations. Deletions events are sometimes shared by several sequences because they occurred before transposition. All these redundant deletions were counted only once.

Shorter copies (less than 1000 bp) with evidence of transposition (at least 2 copies with TIRs, bordered by different flanking regions) were considered as MITEs. MITE classification relied on homology with longer MLEs (in the TIRs and internal sequences) and on the breakpoints of deletion/rearrangements. In this analysis, MITEs with no internal homology could not be retrieved.

### Analysis of amplification dynamics

Species-specific amplification dynamics of single lineages were inferred using a new method based on the phylogenetic tree node distributions over time. This method relies on the topology of the tree and offers a visualization of the variation in transposition rate over time. More details are available in Le Rouzic, Payen, Hua-Van [34]. The trees were rooted with an outgroup corresponding to the consensus sequence of the closest MLE lineage. All trees were reconstructed using FastTree 2.1 [68]. All insertions were removed from the sequence dataset.

### Phylogenetic analyses

The phylogenetic analysis of transposases was performed with MrBayes 3.2.1 [69] using the transposase derived from the consensus of each lineage along with the *MLE* proteins used in the BLASTP searches. For this analysis, we used the amino acid substitution model WAG + G, suggested as the best model by ProtTest 3 [70]. Two million generations were evaluated, sampling the most probable tree every 100 generations and burning 25% of those. Sequences used as outgroups were *Bmmar1* from *Bombyx mori,* a *Tc1-mariner* element from the *maT* (DD37D) family and *Tvmar1* from *Trichomonas vaginalis*. The resulting tree can be acessed at TreeBASE repository [71].

### Insertion sites

For each subfamily, the percentage of T or A at the 10 positions surrounding the TA insertion site was computed and compared to the percentage found for all TAs in the 20 genomes. For each *mariner* lineage, a consensus derived from the immediate flanking sequence was calculated with WebLogo [72].

### Availability of supporting data

The genome sequences of the *Drosophila* species can be accessed at FlyBase (http://flybase.org/) or at NCBI (http://www.ncbi.nlm.nih.gov/). All mariner copies can be retrieved using their positions given in Additional file 2: Table S2.

## Electronic supplementary material

Additional file 1: Table S1: Description of all lineages identified in the 20 species. (PDF 80 KB)

Additional file 2: Table S2: Supercontig locations of all *mariner* copies identified in this study. Positions refer to start and end of regions reported as similar to the consensus sequence after MEGABLAST analysis. They may sometimes slightly differ from positions after manual curation. (XLSX 163 KB)

Additional file 3: Figure S1: Open Reading Frames and TIRs. For each lineage, (+ +) indicated the presence of uninterrupted ORF in both copies and consensus sequence, (− +) indicated the presence of uninterrupted ORF in the consensus sequence only. TIR consensus are reported on the right. (PDF 310 KB)

Additional file 4: Table S3: Description of MITE sublineages. (PDF 57 KB)

Additional file 5: Figure S2: Violin pots depicting the distribution of copies relative to genes for *Dromar5* and *Dromar11* in *D. Eugracilis.* Significant differences (Kolgomorov-Smirnov test) are indicated. M: MITE lineage, FL: full-length lineage. n: copy number (percentage of copies located on genes-containing contigs). (PDF 101 KB)

Additional file 6: Table S4: Sequence evolution characteristics in *mariner* lineages. (XLSX 54 KB)

Additional file 7: Figure S3: Analysis of the trinucleotide preference in 5′ (A) and 3′ (B) of the insertion TA site for the *mellifera* and *drosophila* subfamilies. Only trinucleotides found more than 5 times in the data are presented. (PDF 151 KB)

Additional file 8: Figure S4: Consensus analysis of the region surrounding the insertion sites of different lineages, using WebLogo [71]. The duplicated target TA is present on each site (central TAs). The elements have been removed. The numbers into parentheses indicated the number of 5′ and 3′ flanking regions analysed. (PDF 291 KB)
