# Supplementary material for: Genomic landscape and evolutionary dynamics of mariner transposable elements within the Drosophila genus
Source: BMC Genomics. 2014 Aug 27;15(1):727. doi: 10.1186/1471-2164-15-727 (PMC4161770; doi:10.1186/1471-2164-15-727)
Supplement: Supplementary file 1 — Additional file 1: Table S1: Description of all lineages identified in the 20 species. (PDF 80 KB) [file 12864_2014_6424_MOESM1_ESM.pdf]

Table S1. Description of all lineages identified in the 20 species.

Subfamilies	Lineages	Total copy number	Independent copy number	Copies with ORF	MITEs			MITE size in bp (M1, M2, M3)
					M1	M2	M3	
mauritiana	Dromar1sim	2	2	1				
mauritiana	Dromar1sec	4	4					
mauritiana	Dromar1yak	5	3	2				
mauritiana	Dromar9ana	11	11					
mauritiana	Dromar9kik	8	8					
mauritiana	Dromar9fic	2	2					
mauritiana	Dromar19yak	4	4					
mauritiana	Dromar19ere	13	13		6			900
mauritiana	Dromar24fic	5	5					
mauritiana	Dromar24eug	10	10		5			550
mauritiana	Dromar24ana	6	5					
mauritiana	Dromar24bip	9	8		8			928
mauritiana	Dromar25fic	2	2					
mauritiana	Dromar25bip	9	4		4			910
mauritiana	Dromar26rho	7	7					
mauritiana	Dromar26ana	2	2		2			560
mauritiana	Dromar26bip	8	7		6			930
mauritiana	Dromar27fic	7	7					
mauritiana	Dromar32fic	4	3		3			905
mellifera	Dromar6yak	17	12	2				
mellifera	Dromar6ere	611	469	72				
mellifera	Dromar6fic	3	3					
mellifera	Dromar6ele	6	6					
mellifera	Dromar6rho	65	59					
mellifera	Dromar6ana	40	35	3				
mellifera	Dromar6bip	32	24	5				
mellifera	Dromar13fic	67	63					
mellifera	Dromar13eug	11	11					
mellifera	Dromar13rho	19	15					
mellifera	Dromar13kik	14	14					
mellifera	Dromar13ana	16	15					
mellifera	Dromar13bip	8	8					
mellifera	Dromar5fic	37	35	2				
mellifera	Dromar5eug	184	167	1	103			526
mellifera	Dromar5bia	2	2					
mellifera	Dromar5tak	5	5					
mellifera	Dromar5ele	13	11					
mellifera	Dromar5rho	10	9					
mellifera	Dromar5kik	19	15					
mellifera	Dromar5ana	69	60					
mellifera	Dromar5bip	16	15					
mellifera	Dromar17ele	1	1					
mellifera	Dromar17fic	1	1					
mellifera	Dromar17moj	52	42	29				
mellifera	Dromar14mel	7	4					
mellifera	Dromar14sim	2	2					

Subfamilies	Lineages	Total copy number	Independent copy number	Copies with ORF	MITEs			MITE size in bp (M1, M2, M3)
					M1	M2	M3	
mellifera	Dromar14sec	3	1					
mellifera	Dromar14rjo	1	1					
mellifera	Dromar29fic	95	69	1				
mellifera	Dromar29tak	3	3					
irritans	Dromar15ana	17	16					
irritans	Dromar16fic	10	9	3				
irritans	Dromar16rho	5	3					
irritans	Dromar16eug	1	1					
irritans	Dromar16tak	5	2	1				
irritans	Dromar16kik	21	19		19			930
irritans	Dromar16ana	11	11	1				
irritans	Dromar18bip	1	0					
irritans	Dromar18yak	118	68	39				
irritans	Dromar10fic	2	2					
irritans	Dromar10eug	13	10					
irritans	Dromar10bia	80	75					
irritans	Dromar10tak	4	4					
irritans	Dromar10rho	76	68					
irritans	Dromar10kik	7	6					
irritans	Dromar10ana	32	31					
irritans	Dromar10bip	3	3					
irritans	Dromar10pse	1	1					
irritans	Dromar10per	1	1					
irritans	Dromar10wil	11	11					
irritans	Dromar21ana	12	11					
irritans	Dromar21fic	5	4					
irritans	Dromar21tak	4	3					
irritans	Dromar21ele	3	3	1				
irritans	Dromar21kik	14	11					
irritans	Dromar23rho	38	33					
irritans	Dromar23ana	6	4					
irritans	Dromar33kik	11	11		6			910
irritans	Dromar33bip	23	21					
irritans	Dromar34fic	2	2					
irritans	Dromar34tak	5	5					
irritans	Dromar34kik	6	6					
irritans	Dromar34bip	2	2					
irritans	Dromar35fic	2	2					
vertumnana	Dromar3pse	30	28					
vertumnana	Dromar3per	40	30					
vertumnana	Dromar2yak	6	6					
vertumnana	Dromar2ere	1	1					
vertumnana	Dromar2eug	4	3					
vertumnana	Dromar2tak	4	3					
vertumnana	Dromar2rho	11	9					
vertumnana	Dromar2ana	100	88					
vertumnana	Dromar2bip	9	9					
vertumnana	Dromar12fic	5	5					

Subfamilies	Lineages	Total copy number	Independent copy number	Copies with ORF	MITEs			MITE size in bp (M1, M2, M3)
					M1	M2	M3	
vertumnana	Dromar12bip	3	3					
vertumnana	Dromar12ana	20	17					
drosophila	Dromar4sec	4	4					
drosophila	Dromar4yak	3	3					
drosophila	Dromar4ere	13	13					
drosophila	Dromar4ana	65	50					
drosophila	Dromar4tak	6	6					
drosophila	Dromar4bia	109	97					
drosophila	Dromar4ele	5	5					
drosophila	Dromar4rho	4	4					
drosophila	Dromar4kik	13	9					
drosophila	Dromar4bip	4	4					
drosophila	Dromar7ere	50	41		14	18		970, 954
drosophila	Dromar8ana	1	1					
drosophila	Dromar8ere	2	1					
drosophila	Dromar8fic	84	83	1	34			959
drosophila	Dromar8gri	90	51	16				
drosophila	Dromar11eug	487	440	9	88	314	5	947, 467,934
drosophila	Dromar11bia	199	157	2				
drosophila	Dromar11ele	4	2					
drosophila	Dromar11gri	1	0					
drosophila	Dromar11fic	1	1					
drosophila	Dromar11rho	13	6					
drosophila	Dromar11ana	26	22		22			950
drosophila	Dromar11bip	46	42	1				
drosophila	Dromar20ere	10	8					
drosophila	Dromar22fic	4	4		4			928
drosophila	Dromar22ele	5	5		2	2		928-944
drosophila	Dromar22rho	4	3		1			927
drosophila	Dromar22ana	15	13		13			918
drosophila	Dromar22bip	9	5		4			913-939
drosophila	Dromar28fic	9	9		9			910
drosophila	Dromar30fic	32	21		19			937
drosophila	Dromar30ele	3	3					
drosophila	Dromar30ana	3	3					
drosophila	Dromar30bip	4	4					
drosophila	Dromar31fic	10	9		9			950
drosophila	Dromar36fic	12	10		5			941
drosophila	Dromar36ele	3	2					
**TOTAL**		**3685**	**3086**	**192**	**386**	**334**	**5**	
