# Supplementary material for: Genomic landscape and evolutionary dynamics of mariner transposable elements within the Drosophila genus
Source: BMC Genomics. 2014 Aug 27;15(1):727. doi: 10.1186/1471-2164-15-727 (PMC4161770; doi:10.1186/1471-2164-15-727)

Figure S1. Open Reading Frames and TIRs. For each lineage, (+ +) indicated the presence of uninterrupted ORF in both copies and consensus sequence, (+) indicated the presence of uninterrupted ORF in the consensus sequence only. TIR consensus are reported on the right.

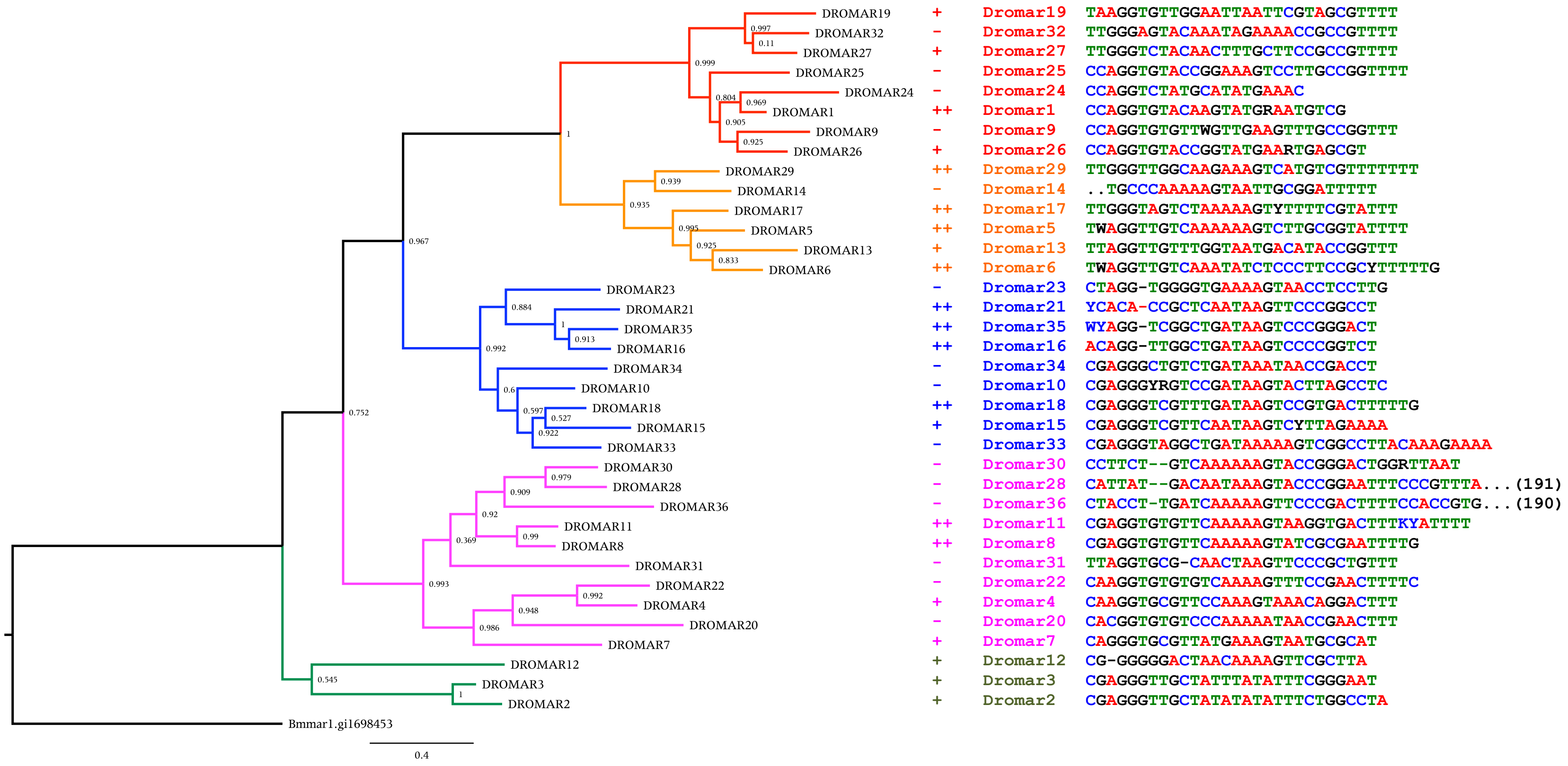

Supplement: Supplementary file 3 — Additional file 3: Figure S1: Open Reading Frames and TIRs. For each lineage, (+ +) indicated the presence of uninterrupted ORF in both copies and consensus sequence, (− +) indicated the presence of uninterrupted ORF in the consensus sequence only. TIR consensus are reported on the right. (PDF 310 KB) [file 12864_2014_6424_MOESM3_ESM.pdf]
