# Supplementary material for: Genomic landscape and evolutionary dynamics of mariner transposable elements within the Drosophila genus
Source: BMC Genomics. 2014 Aug 27;15(1):727. doi: 10.1186/1471-2164-15-727 (PMC4161770; doi:10.1186/1471-2164-15-727)
Supplement: Supplementary file 4 — Additional file 4: Table S3: Description of MITE sublineages. (PDF 57 KB) [file 12864_2014_6424_MOESM4_ESM.pdf]

Table S3. Description of MITE sublineages

Subfamilies	Lineages	MITE Copy Number	Associated Non-MITE Copies	ORF	Size (nt)	TIR size	Mechanism
mauritiana	Dromar19Mere	6	7	0	900	30	internal deletion
mauritiana	Dromar24Meug	5	5	0	550	21	internal deletion
mauritiana	Dromar24Mbip	8	0	0	928	21	internal deletion
mauritiana	Dromar25Mbip	4	0	0	910	31	internal deletion
mauritiana	Dromar26Mana	2	0	0	560	29	internal deletion
mauritiana	Dromar26Mbip	6	1	0	930	29	internal deletion
mauritiana	Dromar32fMic	3	0	0	905	30	internal deletion
mellifera	DromarM5eug	103	64	1	526	28	internal deletion
irritans	Dromar16Mkik	19	0	0	930	27	internal deletion
irritans	Dromar33Mkik	6	5	0	910	39	internal deletion
drosophila	Dromar7M1ere	14	9	0	970	262	rearrangment
drosophila	Dromar7M2ere	18	9	0	954	338	rearrangment
drosophila	Dromar8Mfic	34	49	1	959	32	internal deletion
drosophila	Dromar11M1eug	88	33	9	947	196	rearrangment
drosophila	Dromar11M2eug	314	33	9	467	66	rearrangment
drosophila	Dromar11M3eug	5	33	9	934	243	rearrangment
drosophila	Dromar11Mana	22	0	0	950	30	internal deletion
drosophila	Dromar22Mfic	4	0	0	928	32	internal deletion
drosophila	Dromar22M1ele	2	1	0	928	32	internal deletion
drosophila	Dromar22M2ele	2	1	0	944	32	internal deletion
drosophila	Dromar22Mrho	1	2	0	927	32	internal deletion
drosophila	Dromar22Mana	12	1	0	918	32	internal deletion
drosophila	Dromar22Mbip	4	1	0	913-939	32	internal deletion
drosophila	Dromar28fMic	9	0	0	910	191	rearrangment
drosophila	Dromar30fMic	19	2	0	937	34	internal deletion
drosophila	Dromar31fMic	9	0	0	950	29	internal deletion
drosophila	Dromar36fMic	5	5	0	941	190	rearrangment
