# Supplementary material for: Genomic landscape and evolutionary dynamics of mariner transposable elements within the Drosophila genus
Source: BMC Genomics. 2014 Aug 27;15(1):727. doi: 10.1186/1471-2164-15-727 (PMC4161770; doi:10.1186/1471-2164-15-727)

Figure S2 . Violin pots depicting the distribution of copies relative to genes for *Dromar5* and *Dromar11* in *D. Eugracilis*. Significant differences (Kolgomorov-Smirnov test) are indicated. M: MITE lineage, FL: full-length lineage. n: copy number (percentage of copies located on genes-containing contigs)

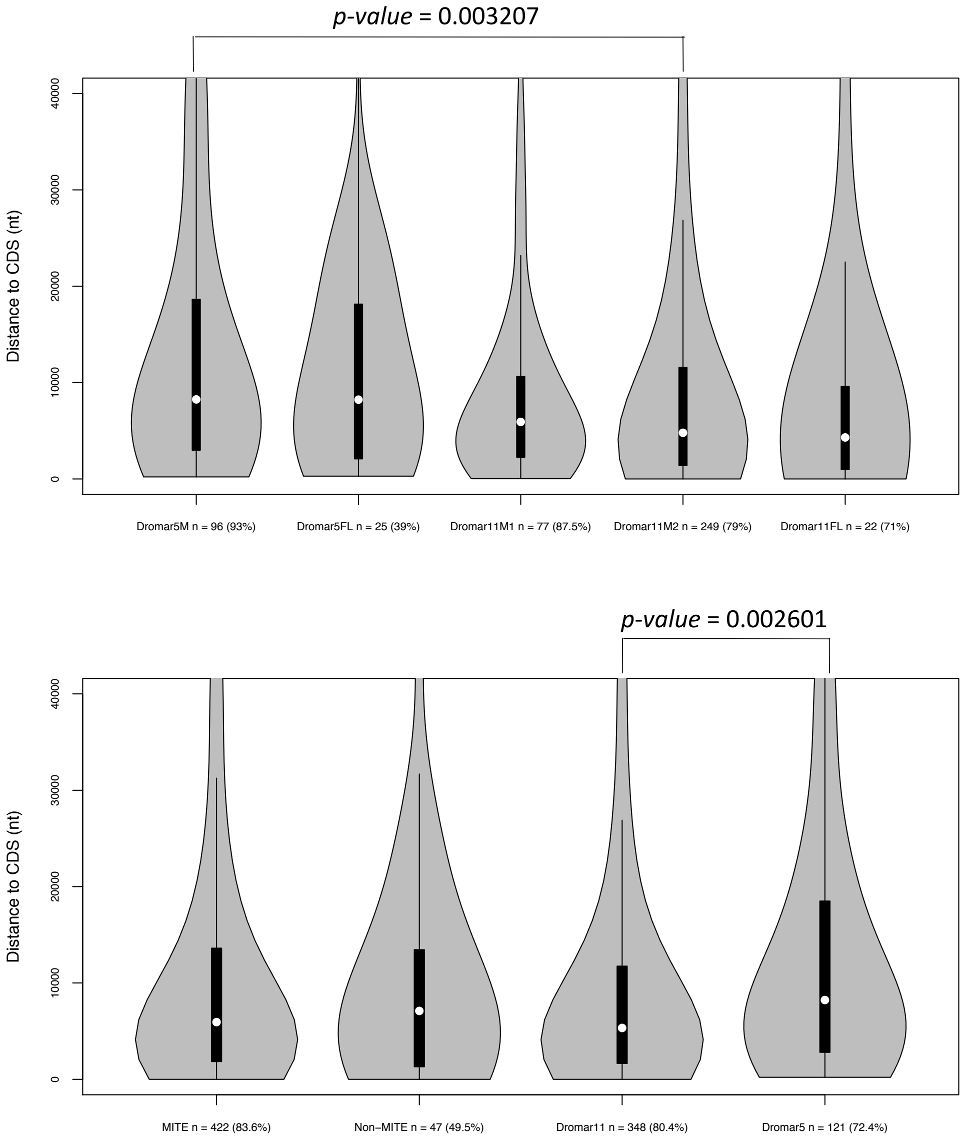

Supplement: Supplementary file 5 — Additional file 5: Figure S2: Violin pots depicting the distribution of copies relative to genes for Dromar5 and Dromar11 in D. Eugracilis. Significant differences (Kolgomorov-Smirnov test) are indicated. M: MITE lineage, FL: full-length lineage. n: copy number (percentage of copies located on genes-containing contigs). (PDF 101 KB) [file 12864_2014_6424_MOESM5_ESM.pdf]
