# Supplementary material for: Genomic landscape and evolutionary dynamics of mariner transposable elements within the Drosophila genus
Source: BMC Genomics. 2014 Aug 27;15(1):727. doi: 10.1186/1471-2164-15-727 (PMC4161770; doi:10.1186/1471-2164-15-727)

Fiigure S3. Analysis of the trinucleotide preference in 5’ (A) and 3’ (B) of the insertion TA site for the *mellifera* and *drosophila* subfamilies. Only trinucleotides found more than 5 times in the data are presented

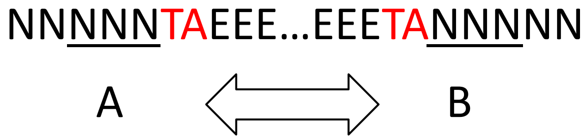

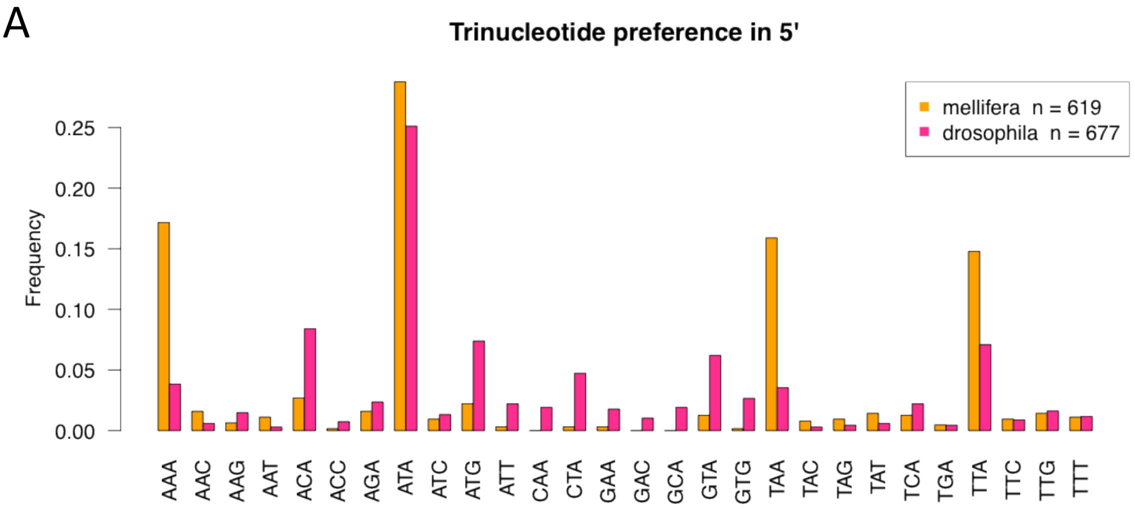

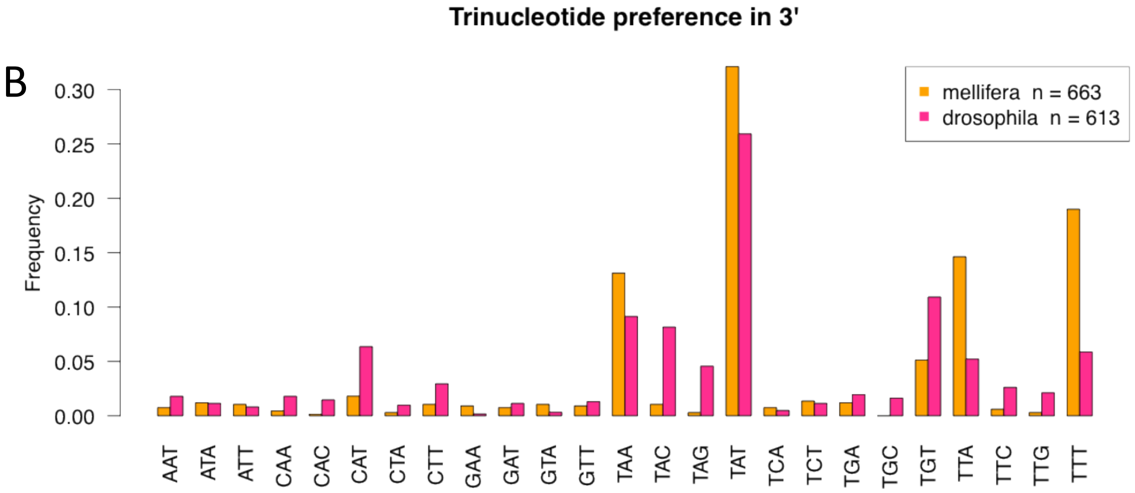

Supplement: Supplementary file 7 — Additional file 7: Figure S3: Analysis of the trinucleotide preference in 5′ (A) and 3′ (B) of the insertion TA site for the mellifera and drosophila subfamilies. Only trinucleotides found more than 5 times in the data are presented. (PDF 151 KB) [file 12864_2014_6424_MOESM7_ESM.pdf]
