# Supplementary material for: Genomic landscape and evolutionary dynamics of mariner transposable elements within the Drosophila genus
Source: BMC Genomics. 2014 Aug 27;15(1):727. doi: 10.1186/1471-2164-15-727 (PMC4161770; doi:10.1186/1471-2164-15-727)

Figure S4. Consensus analysis of the region surrounding the insertion sites of different lineages, using WebLogo (Crooks et al. 2004). The duplicated target TA is present on each site (central TAs). The elements have been removed. The numbers into parentheses indicated the number of 5' and 3' flanking regions analysed.

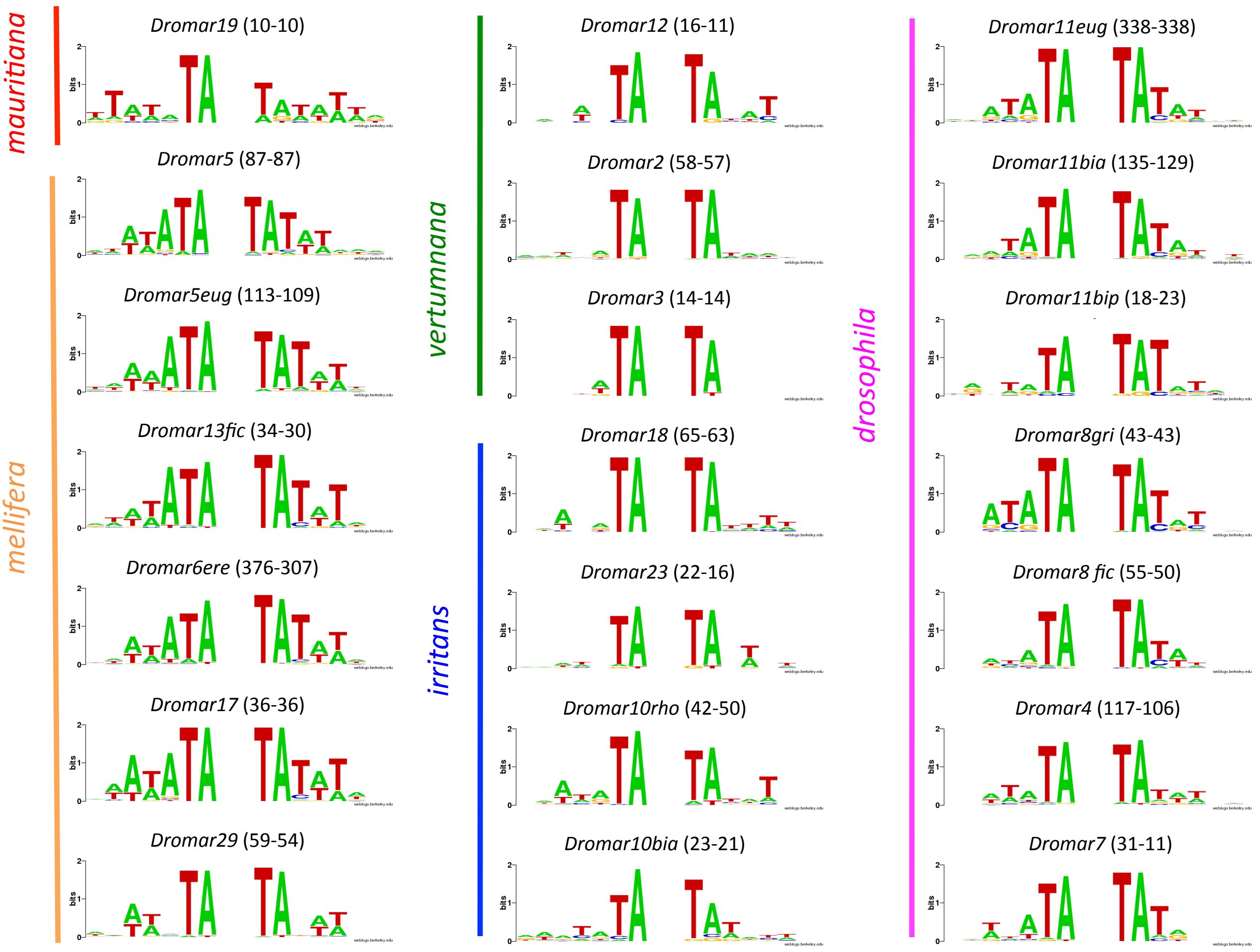

Supplement: Supplementary file 8 — Additional file 8: Figure S4: Consensus analysis of the region surrounding the insertion sites of different lineages, using WebLogo [71]. The duplicated target TA is present on each site (central TAs). The elements have been removed. The numbers into parentheses indicated the number of 5′ and 3′ flanking regions analysed. (PDF 291 KB) [file 12864_2014_6424_MOESM8_ESM.pdf]
